# Supplementary material for: Tumorigenicity decrease in Bcl-xL deficient MDCK cells ensuring the safety for influenza vaccine production
Source: PLoS One. 2024 Dec 16;19(12):e0311069. doi: 10.1371/journal.pone.0311069 (PMC11649150; doi:10.1371/journal.pone.0311069)
Supplement: S1 File — (ZIP) [file pone.0311069.s002.zip › S2_File/4_KOG_enrichment/enrichmap/B_vs_A.down_KOG_enrichment_map.pdf]

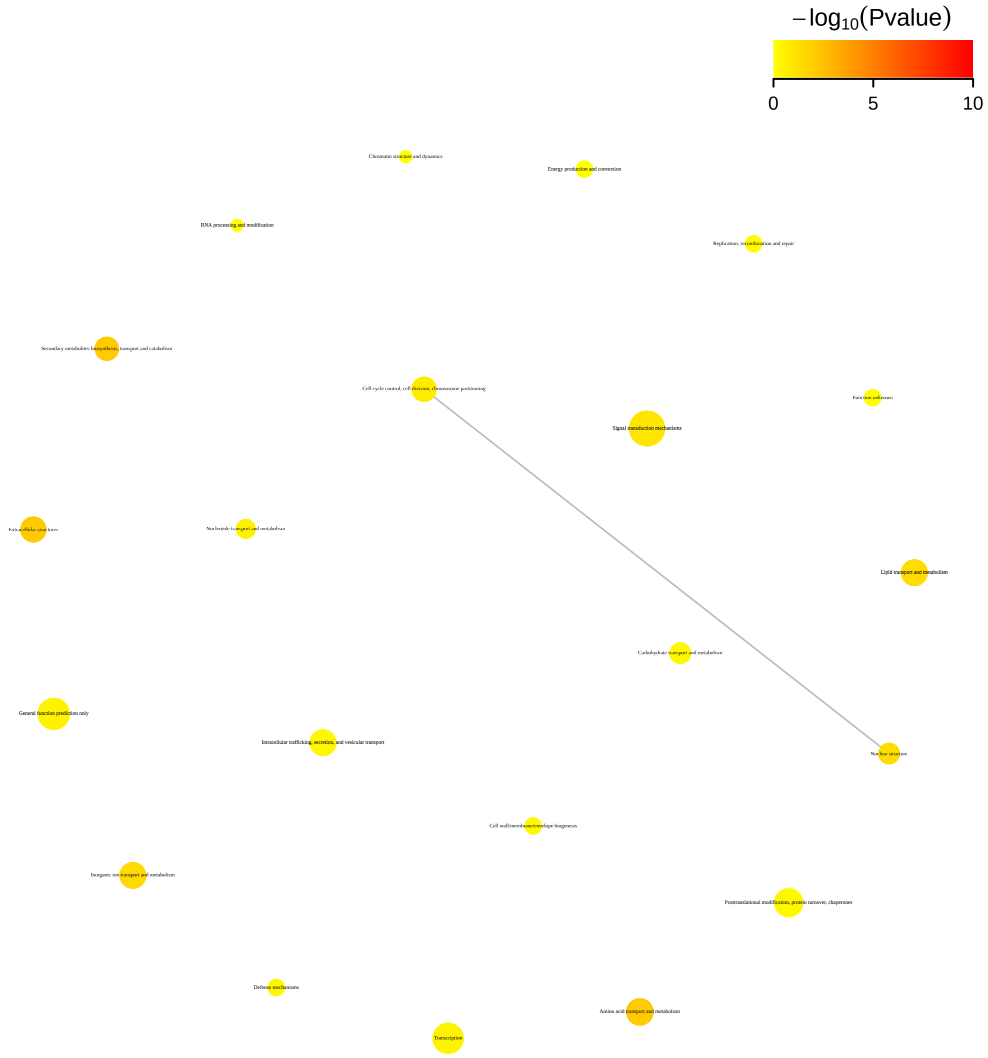
$-\log_{10}(\text{Pvalue})$
0
5
10
Chromatin structure and dynamics
Energy production and conversion
RNA processing and modification
Replication, recombination and repair
Secondary metabolites biosynthesis, transport and catabolism
Cell cycle control, cell division, chromosome partitioning
Function unknown
Signal transduction mechanisms
Extracellular structures
Nucleotide transport and metabolism
Lipid transport and metabolism
Carbohydrate transport and metabolism
General function prediction only
Intracellular trafficking, secretion, and vesicular transport
Nuclear structure
Cell wall/membrane/envelope biogenesis
Inorganic ion transport and metabolism
Posttranslational modification, protein turnover, chaperones
Defense mechanisms
Amino acid transport and metabolism
Transcription
